# Correlation Between Epicardial Adipose Tissue and PET Cardiac Perfusion: A Systematic Review

**DOI:** 10.3390/medsci14020194

**Published:** 2026-04-11

**Authors:** Francesco Dondi, Pietro Bellini, Mattia Bertoli, Gian Luca Viganò, Roberto Rinaldi, Luca Camoni, Michela Cossandi, Enrico Vizzardi, Carlo Mario Lombardi, Francesco Bertagna

**Affiliations:** 1 Nuclear Medicine, ASST Spedali Civili di Brescia and University of Brescia, 25123 Brescia, Italy; 2 Nuclear Medicine, ASST Spedali Civili di Brescia, 25123 Brescia, Italymattia.bertoli@asst-spedalicivili.it (M.B.); roberto.rinaldi@asst-spedalicivili.it (R.R.); michela.cossandi@asst-spedalicivili.it (M.C.); 3 Clinical Engineering, ASST Spedali Civili di Brescia, 25123 Brescia, Italy; gianluca.vigano@asst-spedalicivili.it; 4 Allied Health and Social Care Professions Directorate, ASST Spedali Civili di Brescia and University of Brescia, 25123 Brescia, Italy; luca.camoni@unibs.it; 5 Institute of Cardiology, ASST Garda, Department of Medical and Surgical Specialties, Radiological Sciences, and Public Health, University of Brescia, 25123 Brescia, Italy; enrico.vizzardi@unibs.it; 6 Institute of Cardiology, ASST Spedali Civili di Brescia, Department of Medical and Surgical Specialties, Radiological Sciences, and Public Health, University of Brescia, 25123 Brescia, Italy; carlo.lombardi@unibs.it

**Keywords:** epicardial adipose tissue, EAT, positron emission tomography, PET, myocardial perfusion imaging, coronary artery disease

## Abstract

**Background**: Evidence on the presence of an association between epicardial adipose tissue (EAT) and myocardial perfusion imaging (MPI) as assessed by positron emission tomography (PET) has been reported. This systematic review aimed to synthesize the existing literature investigating this topic. **Methods**: A comprehensive and systematic search of the PubMed/MEDLINE, Scopus, and Embase databases was performed to identify published studies investigating the association between EAT and myocardial perfusion assessed by PET imaging. Eligible studies included original research articles evaluating EAT and reporting PET MPI outcomes. Data regarding the study design, patient characteristics, imaging protocols, and main findings were extracted and qualitatively analyzed. **Results**: Ten studies were included in the final analysis. Overall, most studies demonstrated a significant association between increased EAT and impaired myocardial perfusion on PET imaging. In several studies, EAT remained an independent predictor of abnormal PET MPI after adjustment for traditional clinical risk factors. Nonetheless, important methodological differences among studies were observed, including heterogeneity in EAT measurement techniques, quantification methods, and PET tracers used for MPI evaluation, which limit the generalizability of these findings. **Conclusions**: This systematic review seems to suggest a potential association between increased EAT and impaired myocardial perfusion, as assessed by PET. However, significant methodological heterogeneity across the available studies—including differences in EAT quantification, PET protocols, and tracer selection—limits the strength of this conclusion. Standardized imaging protocols and larger, prospective, multicenter studies are required to validate this relationship, determine its incremental prognostic value, and evaluate its potential for integration into routine clinical risk stratification pathways.

## 1. Introduction

Epicardial adipose tissue (EAT) is a visceral fat depot located between the myocardium and the visceral layer of the pericardium. Beyond its role as an energy reservoir and fat storehouse for the heart, EAT is now recognized as a metabolically active tissue that secretes a wide range of pro-inflammatory cytokines, adipokines, and bioactive molecules, potentially influencing the coronary artery physiology and myocardial function through paracrine and vasocrine mechanisms [1]. Growing evidence suggests that increased EAT volume or thickness is associated with coronary artery disease (CAD), endothelial dysfunction, and adverse cardiovascular outcomes [2,3,4,5,6,7,8]. In particular, it has been underlined that EAT may be helpful for the estimation of the pretest probability of CAD over traditional risk factors and the coronary calcium score (CCS) [9].

Myocardial perfusion imaging (MPI) is currently a fundamental diagnostic modality for the evaluation and the management of CAD patients [10,11,12,13]. Positron emission tomography (PET) represents a well-established, highly accurate non-invasive technique for the assessment of stress and rest myocardial blood flow (MBF), myocardial flow reserve (MFR) and coronary microvascular function with great accuracy [14]. These parameters allow PET to detect ischemia and diffuse atherosclerosis and microvascular dysfunction, even in the absence of significant epicardial coronary stenosis [15,16,17]. Moreover, this imaging modality, when performed with different radiopharmaceuticals, has demonstrated its role in the evaluation of different conditions that can affect the cardiovascular system, such as atherosclerotic plaques and their inflammation processes, sarcoidosis or endocarditis [18,19,20].

In recent years, several studies have explored the relationship between EAT and myocardial perfusion assessed by PET, hypothesizing that excess epicardial fat may impair coronary microcirculation and myocardial perfusion. Therefore, the aim of this systematic review is to comprehensively evaluate and synthesize the existing literature investigating the association between EAT and MPI as assessed by PET imaging. From a nuclear medicine perspective, investigating this correlation is particularly pertinent. In clinical practice, myocardial perfusion PET/computed tomography (CT) protocols routinely include a non-contrast computed tomography scan for attenuation correction. This same CT dataset can be repurposed for the quantification of EAT without requiring additional image acquisition, radiation exposure, or patient time. Therefore, elucidating a robust link between EAT and the perfusion parameters could unlock a readily available, incremental biomarker for refining cardiovascular risk assessment within the existing imaging workflows.

## 2. Materials and Methods

The systematic review was designed and performed according to the “Preferred Reporting Items for a Systematic Review and Meta-Analysis” (PRISMA 2020 statement), which was used as a guide in its development. The complete PRISMA checklist is available in the Supplementary Materials. The review has not been registered in PROSPERO.

### 2.1. Search Strategy

A comprehensive literature search was conducted in three major databases (PubMed/MEDLINE, Scopus, and Embase) to identify relevant articles assessing the relationship between EAT and MPI evaluated with PET imaging. The search strategy used the following query: (“PET” OR “PET/CT” OR “positron emission tomography”) AND “epicardial adipose tissue”.

No start date restriction was applied, and the search was updated until 30 January 2026. Only articles published in English were considered. Preclinical studies, conference abstracts, reviews, editorials, and case reports were excluded. In addition, the reference lists of all included articles were manually screened to identify additional relevant studies that may have been missed during the database search. This approach ensured a thorough and systematic identification of the literature while minimizing the risk of omitting pertinent studies.

### 2.2. Study Selection

Two researchers (F.D. and F.B.) independently screened the titles and abstracts of the articles retrieved with the database search. The same two researchers independently reviewed the full-text versions of the identified articles to determine their eligibility for inclusion in the review. In the case of any discrepancies between the two reviewers, these were resolved by consensus; however, no studies required such a consensus. Clinical studies analyzing the presence of possible correlations between EAT and MPI assessed with PET/CT imaging were deemed eligible for inclusion in this systematic review.

### 2.3. Quality Assessment

The quality assessment of these studies, including the risk of bias and applicability concerns, was carried out using the Quality Assessment of Diagnostic Accuracy Studies version 2 (QUADAS-2) evaluation [21]. This quality evaluation was performed independently by the two reviewers, and discrepancies were again resolved by consensus.

### 2.4. Data Extraction

The same two reviewers previously mentioned independently evaluated the retrieved studies to collect relevant information to be included in the review. For each study, data concerning the basic information of the study, such as the first author’s name, year of publication, country of origin, design of the study, radiopharmaceutical used, and number of patients, were collected. Furthermore, information about the type of PET tomograph used, the activity of the injected tracer, and the main results were also obtained. The setting and endpoint of the studies were also collected. Specific details on the methodology used for EAT quantification in each study were systematically collected, including the type of measurement (volume, thickness, or indexed volume), the software platform, the defined Hounsfield Unit (HU) attenuation thresholds, and whether the process was manual or semi-automated. The main findings of the articles included in this review are reported in the Results section.

## 3. Results

### 3.1. Literature Search

**Figure 1:**
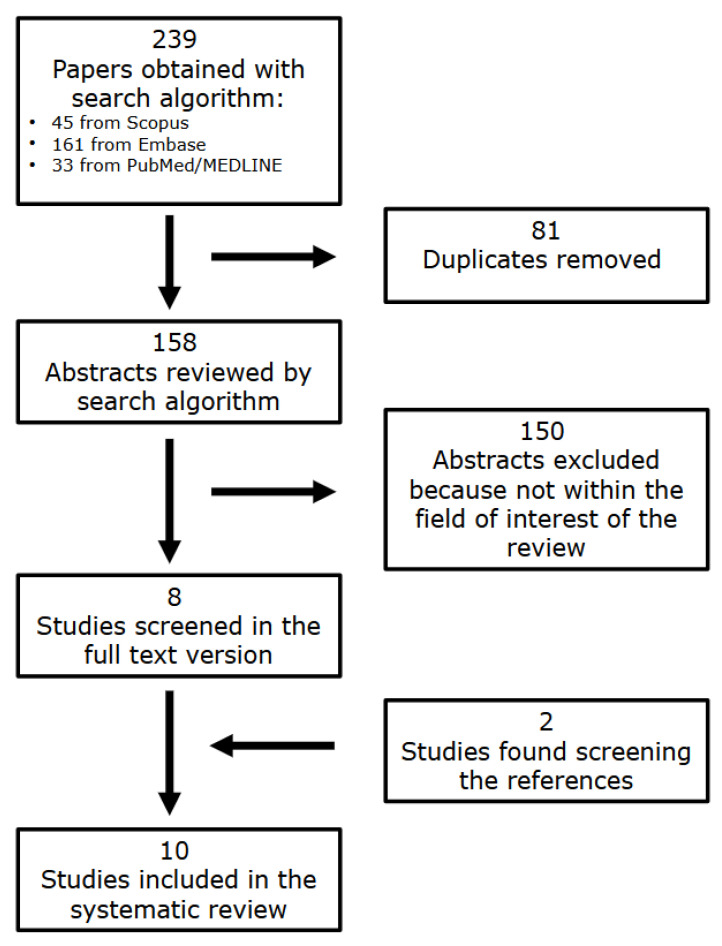
Flowchart of the research of eligible studies evaluating the correlation between EAT and PET MPI.

The literature search retrieved a total of 158 articles. After reviewing the titles and abstracts, 150 studies were excluded by applying the exclusion criteria and consequently, eight studies analyzing the relationship between EAT and MPI assessed with PET imaging were selected and retrieved in the full-text version [22,23,24,25,26,27,28,29]. Two additional studies were identified after analyzing the reference lists of these articles [30,31]. Therefore, a total number of 10 studies were included in the review [22,23,24,25,26,27,28,29,30,31] (Figure 1).

**Figure 2:**
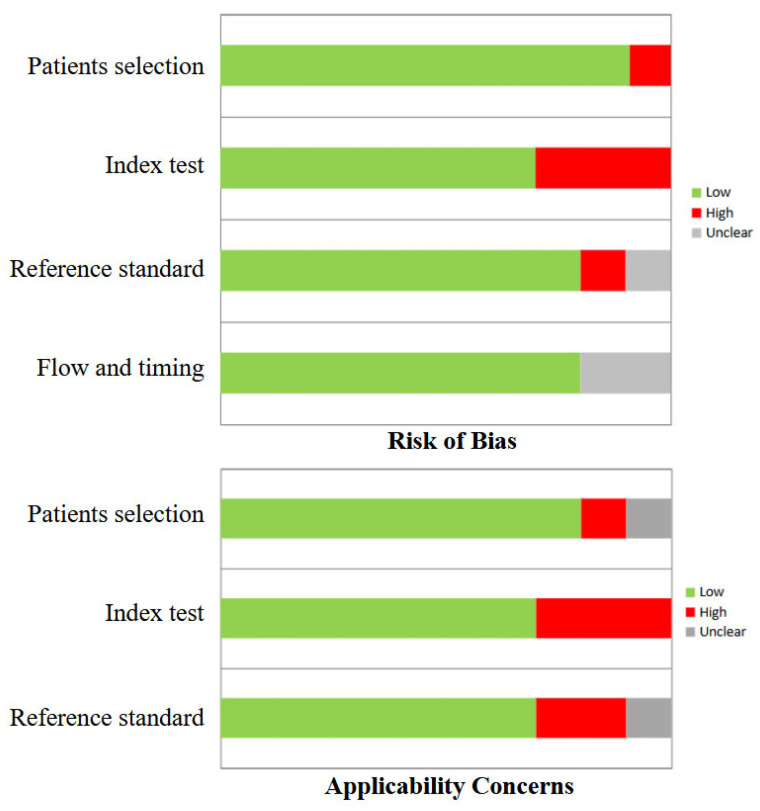
QUADAS-2 quality assessment for risk of bias and applicability concerns for the studies included in the review.

In general, the quality assessment using QUADAS-2 evaluation underlined the presence of a low risk of bias in most domains for all studies included in the review. The index test and reference standard were characterized by a slightly higher risk of bias (Figure 2).

In this setting, it is worth underlining that a higher variability between PET/CT protocols, stress and rest protocols and radiotracers used in different studies was present. Furthermore, different modalities and parameters among studies for the extraction of EAT-derived parameters were reported. Consequently, a direct comparative synthesis of quantitative results across studies was not feasible. The observed variability in PET tracer kinetics, EAT measurement techniques, and analytical approaches constitutes a fundamental source of clinical and methodological heterogeneity. This must be critically considered when interpreting the aggregated findings of this review, as it precludes a formal meta-analysis and suggests that the potential associations should be viewed within the specific context of each study’s methodology.

All the studies included in the systematic review had a retrospective design and were performed on PET/CT tomographs [22,23,24,25,26,27,28,29,30,31]. In terms of radiopharmaceuticals used, seven studies were performed using [82Rb] [22,23,24,26,27,30,31], one with [82Rb] and [13N] NH_3_ [28], one with [13N] NH_3_ [29] and one with [15O] H_2_O [25]. The main characteristics of the studies and their results are briefly presented in Table 1, Table 2 and Table 3.

### 3.2. Analysis of Heterogeneity Among Studies

The included studies demonstrated substantial heterogeneity across several methodological and clinical dimensions. The study populations varied in terms of sample size, age, and metabolic risk profiles and, while most of them focused on CAD-naïve subjects, some papers also included patients with a previous history of CAD. All studies employed a retrospective design, and most were single-center, which may limit generalizability. PET imaging protocols differed between studies, with variations in radiotracers ([82Rb], [13N] NH_3_, [15O] H_2_O), stress/rest protocols, and scanner models. Similarly, EAT quantification was performed using diverse metrics (volume, indexed volume, thickness, and density), with different measurement techniques (manual vs. semi-automated) and varying HU thresholds. 

The studies included in the present review reported adjustment for potential confounders, with wide variability in the factors included, such as age, sex, body mass index (BMI), diabetes, and CCS. Moreover, different endpoints were considered among the papers; for example, in some of them, the presence of ischemia or MFR were used, while others focused on the prognostic value of EAT. These differences collectively contribute to clinical and methodological heterogeneity, precluding direct quantitative synthesis and necessitating careful interpretation of pooled findings.

### 3.3. Correlation Between EAT and PET/CT Imaging

The correlation between EAT and MPI PET/CT findings was explored in different papers in several settings and, in most cases, the EAT volume was evaluated. In this scenario, it has been reported that the EAT volume increased significantly from the control group to patients with mild-moderate and severe ischemia (96.9, 124.5 and 143.9 cm^3^, respectively, *p* < 0.01 for both ischemia groups vs. controls) [22]. An interesting paper by Karohl et al. [24] revealed that the EAT volume was higher in patients with perfusion defects compared to those with normal perfusion in a group of subjects that performed [82Rb] MPI prior to kidney transplantation (*p* < 0.001). Moreover, a positive correlation between coronary microvascular resistance (a ratio between mean arterial pressure and hyperemic myocardial blood flow; CMVR) and EAT volume was demonstrated in obese patients who were free from coronary obstruction (r 0.25, *p* 0.001) [25]. When focusing on nonobese subjects or using MFR as a dependent variable, the EAT volume had no significant role as a predictor, allowing us to suggest that this parameter did not provide incremental information on coronary microvascular function beyond traditional risk factors [25]. Despite previous reports, a single interesting paper that investigated deep learning-quantified body composition from [82Rb] or [13N] NH_3_ PET/CT and cardiovascular outcomes, revealed no significant correlations for the EAT volume or density with MFR [28].

Patients with both ischemia and stenosis had significantly higher EAT indexed to their body-surface area (EATi) than subjects without these conditions (64.6 ± 20.6 vs. 49.7 ± 14.2 cm^3^/m^2^, *p* < 0.001). Moreover, the introduction of this parameter in the work-up of patients had a trend towards increased performances for the prediction of concurrent myocardial ischemia and obstructive stenosis over age, gender, chest pain and high CCS (AUC 0.73 vs. 0.65, *p* 0.09); therefore, it was a significant predictor of these conditions [odds ratio (OR) 6.18, 95% confidence interval (CI) 1.73–22.01, *p* 0.005] [30]. Furthermore, it has been reported that subjects with impaired MFR had higher EATi (63.1 ± 20.4 vs. 51.3 ± 14.1 cm^3^/m^2^, *p* 0.003) than those with normal values and that this parameter was the only independent predictor of impaired MFR [odds ratio (OR) 7.39, *p* 0.002] in a specific cohort without any history of prior coronary artery disease [31].

Some papers also investigated and assessed the EAT thickness, revealing that this parameter, in addition to the EAT volume, was higher in patients with an impaired MFR compared to those with normal values (6.7 ± 1.6 mm vs. 4.4 ± 1.0 mm, *p* < 0.001 and 119.0 ± 25.3 cm^3^ vs. 105.8 ± 30.5 cm^3^, *p* < 0.04, respectively). Furthermore, the EAT thickness had a stronger negative correlation with MFR than the EAT volume and CCS (r −0.78 vs. r −0.25 and ρ −0.32, *p* < 0.001), as it was the only parameter that was independently associated with impaired MFR (OR 20.7, 95% CI 4.9–87.9, *p* < 0.001) [23]. These insights were also confirmed when considering the CAD history of the patients, with the EAT volume and EAT thickness being reported as significantly different between subjects with normal and impaired MFR in the total cohort (*p* 0.001 and 0.005, respectively), but also dividing the patients on the presence of previous CAD (*p* 0.017 and 0.038, respectively, for patients without CAD; *p* 0.010 and 0.007, respectively, for subjects with CAD) [29]. Interestingly, the EAT volume was an independent predictor of MFR (*p* 0.008) in the total cohort and in the group of patients without CAD history (*p* 0.038) while, similarly to EAT thickness, it was not an independent predictor of stress MBF [29]. Moreover, it has been reported that in subjects with suspected coronary artery disease but normal [82Rb] PET imaging, the EAT volume (*p* < 0.001) and EAT thickness (*p* 0.009) were higher in those with impaired MFR, while only age (*p* 0.002), heart rate reserve (*p* 0.021) and EAT volume (*p* 0.043) were independently associated with reduced perfusion [26].

Some papers made a direct comparison of the value of EAT and CCS in the prediction of impaired coronary function. In patients with a value of 0 for CCS, a significant relationship between the EAT volume and MFR (*p* 0.014) was observed [26]. In this setting, superior performance of the EAT thickness compared to the EAT volume and CCS in detecting impaired MFR were demonstrated (AUC 0.945 vs. 0.625, *p* < 0.001 and AUC 0.945 vs. 0.710, *p* < 0.006 respectively) [23]. Moreover, it has been reported that the EAT volume had a better correlation with the presence of ischemia than CCS (r 0.47 and 0.28 respectively, *p* < 0.01, AUC 0.764 and 0.629 respectively, *p* 0.04) and that it was, in contrast with CCS, a significant predictor of ischemia after adjusting for age, sex and BMI (*p* < 0.01) [22]. Interestingly, it has also been suggested that the combination of both these parameters did not improve the diagnostic ability of EAT [22]. Moreover, in a cohort of patients waiting for kidney transplantation, the EAT volume (*p* 0.008) and CCS (*p* 0.005) were independent predictors for abnormal [82Rb] PET/CT imaging [24]. In the same cohort, the EAT volume added incremental diagnostic value and discrimination power to a model built by including age, CCS and DM [AUC from 0.73 (95% CI 0.64–0.81) to 0.76 (95% CI 0.68–0.84); *p* 0.02] [24]. An interesting paper performed in a group of CAD-naïve patients proposed a machine learning risk score encompassing different clinico-pathological factors, also including EATi and CCS, that revealed an improvement of risk reclassification for impaired MFR (integrated discrimination improvement compared to EATi 0.22, *p* 0.002) [31]. Interestingly, it has also been reported that the EAT volume (β 0.14, *p* < 0.001) and CCS (β 0.01, *p* < 0.01) were independent predictors of CMVR in obese patients after a BMI-based division [25]. Furthermore, abnormalities in the density of all adipose tissue considered were associated with death or myocardial infarction in a cohort encompassing both CAD-naïve subjects and patients with previous CAD, even after the correction for CCS, MFR, age, sex and clinical risk factors such as BMI (*p* < 0.001 in all the cases for EAT density) [28]. Lastly, an interesting paper by Zobel et al. [27] assessed the relation of cardiac adipose tissue (the sum of EAT and pericardial fat) to CCS and myocardial microvascular function in [82Rb] PET/CT imaging in patients with type I and type II diabetes, revealing that the first was comparable in the controls and type I diabetes subjects, while it was significantly higher in type II patients compared to the controls (*p* < 0.001) and type I subjects (*p* < 0.001); these differences remained significant, even after adjusting for age, sex, BMI and urinary albumin excretion (*p* ≤ 0.02). Moreover, cardiac adipose tissue was positively associated with BMI in the controls and diabetic subjects (*p* ≤ 0.02). In the controls, this parameter was positively associated with CCS (*p* 0.008) and negatively associated with MFR (*p* 0.005); however, no significant association was reported in patients with diabetes.

## 4. Discussion

This systematic review summarizes the current evidence on the relationship between EAT and coronary vascular function assessed by MPI PET imaging. Overall, the included studies consistently indicate that increased EAT quantified as volume, indexed volume or thickness may be associated with myocardial ischemia, impaired MFR and coronary microvascular dysfunction. Moreover, EAT often outperformed or complemented traditional imaging biomarkers such as CCS [22,23,24,25,26,27,28,29,30,31]. However, it is mandatory to underline that relevant heterogeneity exists across study populations, PET tracers, EAT metrics, and analytical approaches, which may partly explain some discordant findings that are present between the different papers included in the present review.

Several studies seemed to demonstrate a robust association between EAT and the presence and severity of myocardial ischemia assessed by PET/CT, suggesting that this parameter may capture both anatomical and functional aspects of coronary disease. In this setting, in some papers it has been proposed that EAT correlated more strongly than CCS and that it was an independent predictor of ischemia, possibly suggesting that EAT may reflect pathophysiological mechanisms beyond coronary calcification [22,28,30]. Furthermore, it has been underlined that the EAT volume progressively increased from the controls to patients with mild–moderate and severe ischemia, suggesting a possible dose–response relationship between these two parameters [22]. In specific high-risk settings, such as kidney transplant candidates, it was further demonstrated that the EAT volume provided incremental diagnostic value beyond CCS and clinical variables for predicting abnormal myocardial perfusion [24]. This finding may highlight the potential clinical utility of EAT quantification in risk stratification, particularly in populations where traditional risk assessment may be suboptimal. From a clinical perspective, a key issue is whether EAT may provide incremental value beyond established markers such as CCS. Overall, even though the evidence presented in the review is still limited, it suggests that EAT may capture aspects of coronary vascular dysfunction that are not fully reflected by coronary calcification, particularly in relation to microvascular impairment and functional abnormalities on PET imaging. This raises the possibility that EAT assessment could complement traditional structural markers by integrating metabolic and inflammatory information. However, the available data remain limited and heterogeneous, largely derived from retrospective and single-center studies, with inconsistent methodologies and scarce outcome-driven validation. Therefore, although EAT appears to be promising as a complementary biomarker, robust prospective studies specifically designed to assess its incremental diagnostic and prognostic value beyond CCS are still needed before its routine clinical implementation can be recommended. Interestingly, it was also suggested that the EAT density, rather than volume, was independently associated with adverse cardiovascular outcomes, despite the lack of correlation with MFR. This finding may support the insight that the compositional and inflammatory characteristics of EAT may be more prognostically relevant than its absolute size [28].

When focusing on coronary microvascular dysfunction, a substantial body of evidence supported a relationship between EAT and this pathological condition, especially when assessed using MFR. This relationship was confirmed in several multivariable models, even after adjustment for age, DM, CCS, and other cardiovascular risk factors. Multiple studies reported a higher EAT volume, EATi, or EAT thickness in patients with reduced MFR, despite normal or non-obstructive coronary arteries [23,26,27,29,31]. Notably, EAT thickness emerged as a particularly strong predictor in some cohorts, showing superior diagnostic performance compared to EAT volume and CCS [23]. Interestingly, it has been reported that the EAT volume predicted impaired MFR, even in patients with zero CCS, supporting the hypothesis that EAT may represent an early marker of coronary vascular dysfunction preceding overt atherosclerosis [26].

The biological mechanisms linking EAT to coronary vascular dysfunction likely involve complex paracrine and vasocrine interactions [1,2,3,4,5,6,7,8]. EAT is a metabolically active tissue that is capable of secreting pro-inflammatory cytokines, adipokines, and reactive oxygen species that may diffuse directly into the adjacent coronary microcirculation, promoting endothelial dysfunction, oxidative stress, and impaired vasodilation [4,5]. However, the pathogenic impact of EAT does not appear to be uniform across different metabolic contexts. In states of systemic metabolic dysfunction, particularly obesity, the inflammatory and lipotoxic profile of EAT may be amplified, enhancing its detrimental local vascular effects and potentially explaining the stronger associations observed in obese individuals. Conversely, in advanced conditions such as overt diabetes, the contribution of EAT may be partially overshadowed by more pervasive and potent mechanisms—including chronic hyperglycemia, advanced glycation end-products, diffuse microvascular remodeling, and autonomic dysfunction—which independently impair coronary flow regulation [32]. This could account for the inconsistent findings across studies and suggests that EAT may act as a disease amplifier in early or intermediate metabolic derangement, whereas in advanced diabetes, its relative contribution becomes more difficult to isolate.

The understanding of the interaction between EAT, obesity and metabolic status remain complex. Some studies revealed that EAT was only associated with increased coronary microvascular resistance in obese patients, while no significant association was observed in non-obese subjects or when MFR was used as the dependent variable, suggesting that the pathological impact of EAT may be amplified in the context of systemic metabolic dysfunction [25]. Additionally, other findings suggested that cardiac adipose tissue was associated with CCS and MFR in the controls, but not in patients with diabetes, indicating that in advanced metabolic disease, other mechanisms may overshadow the contribution of EAT [27]. Conversely, it was also reported that EAT remained an independent predictor of impaired MFR even after accounting for diabetes, underscoring persistent uncertainty regarding effect modification by metabolic status [29,31].

It is possible that the observed correlation between increased EAT and reduced myocardial perfusion on PET/CT may be influenced, at least in part, by imaging artifacts related to CT-based attenuation correction. In hybrid PET/CT imaging, attenuation correction relies on CT-derived density maps to adjust for photon absorption [33]. Because adipose tissue has lower attenuation coefficients compared with soft tissue, substantial fat deposition around the myocardium could theoretically introduce inaccuracies in attenuation maps, particularly if there are misregistration issues between PET and CT datasets due to respiratory or cardiac motion. Such mismatches may lead to over- or under-correction of myocardial activity, potentially mimicking or exaggerating perfusion defects. Therefore, while the association between increased EAT and impaired myocardial perfusion may reflect true pathophysiological mechanisms—such as inflammation or microvascular dysfunction—technical factors related to attenuation correction should also be considered when interpreting these findings.

Beyond PET/CT, cardiac magnetic resonance (CMR) with rest–stress perfusion imaging represents another established non-invasive technique for assessing myocardial perfusion and coronary microvascular function. CMR offers high spatial resolution and multiparametric evaluation of cardiac structure, function, and tissue characteristics without ionizing radiation. Semi-quantitative or quantitative CMR approaches allow for the estimation of the myocardial perfusion reserve, which is conceptually comparable to PET MFR [34,35,36,37]. In addition, CMR techniques enable the quantification of cardiac adipose tissue, including EAT [38,39]. Some insight on the associations between increased EAT and impaired myocardial perfusion reserve or on the value of EAT as an adverse cardiovascular imaging marker, supporting findings from PET-based investigations, have been reported [40]. The data available in the literature are still heterogeneous and limited; therefore, a direct comparison between these two modalities cannot currently be made, and further studies are required in this regard.

From a clinical perspective, the different metrics used to quantify EAT may reflect distinct biological aspects of this tissue. The EAT volume and indexed volume mainly represent the overall burden of epicardial fat and have been most consistently associated with myocardial ischemia and impaired MFR in PET-based studies. The EAT thickness, which is easier to obtain in routine practice, has shown an even stronger association with impaired MFR in some cohorts. Another important metric that is emerging as a significant prognosticator is the EAT density, which can be derived from CT attenuation and may reflect qualitative tissue characteristics, including inflammatory activity, and has been suggested to be closely related to cardiac pathologies and adverse cardiovascular outcomes [41,42,43,44,45]. Therefore, volumetric or thickness measurements may reflect the amount of epicardial fat, while density may provide complementary information on its inflammatory status.

### Limitations

Different limitations affecting this review warrant consideration. First, most of the studies included are retrospective analyses based on single-center settings, an issue that can influence the generalizability of their findings. The retrospective design inherently prevents the establishment of causality between EAT and the perfusion abnormalities and is susceptible to selection bias. Furthermore, it precludes the standardized application of imaging protocols across populations. Therefore, the conclusions should be interpreted with caution, and prospective, multicenter studies are warranted to confirm the robustness and external validity of these observations. Furthermore, confounding was not consistently addressed across studies; adjustments varied widely, and key factors such as age, sex, and comorbidities were sometimes omitted, potentially biasing the observed associations.

Another important point is that different methods to quantify EAT were used across different studies (EAT volume, EAT thickness and EATi), allowing for the capture of distinct aspects of EAT. Interestingly, when a comparison between different EAT-derived parameters has been made, the EAT volume offered better performances in two studies, while the EAT thickness gave better results in a single paper [23,26,29]. Despite that, these parameters are not directly comparable, potentially contributing to heterogeneity across studies. In particular, absolute measurements may be influenced by body size, whereas normalized values aim to reduce this effect. Additionally, the quantification of the EAT volume was performed with different methodologies among different studies, using manual segmentation in some cases, while in other papers, semi-automated methodologies were applied. Moreover, different thresholds were used in terms of HU (e.g., −250 to −30 in some papers, −190 to −30 in others). Furthermore, only three studies assessed the interobserver reproducibility of EAT quantification that, when performed, revealed excellent results [23,25,29]. 

Another important limitation of the review is that the included studies used different tracers to assess myocardial perfusion ([82Rb], [13N] NH_3_ and [15O] H_2_O). In this setting, it is known that different perfusion radiopharmaceuticals have distinct physical and biological properties, which may affect the image quality, quantification and sensitivity to perfusion abnormalities and, most important, the generalizability of the findings proposed in this review [46,47,48,49,50,51,52,53,54,55,56,57,58,59,60,61]. As a result, direct comparison between studies may be limited, and this methodological heterogeneity could contribute to variability in the reported results. 

Another particularly relevant limitation is the scarcity of data linking the association between EAT and PET-derived functional abnormalities to hard clinical outcomes. Although EAT consistently correlates with imaging surrogates such as myocardial ischemia and reduced MFR, robust evidence demonstrating an independent association with major adverse cardiovascular events (e.g., cardiac death, myocardial infarction) is lacking, and follow-up is often limited or unspecified. Therefore, the true incremental prognostic value of EAT beyond the established risk markers remains uncertain. This gap is paramount, as the ultimate clinical value of any biomarker lies in its ability to improve prognostic stratification and guide management decisions beyond what is achievable with existing tools. Moreover, various endpoints in the studies presented here may affect the interpretation of the results. Prospective papers with predefined hard endpoints are needed to determine whether EAT assessment translates into meaningful clinical benefit.

Overall, it must be stated that a formal GRADE assessment was not performed, due to the heterogeneity and limited number of included studies. However, the certainty of evidence can be considered low, given the retrospective design and methodological heterogeneity of the included paper that have been previously underlined. Similarly, publication bias could not be formally assessed due to the limited number and heterogeneity of the included studies that precluded the possibility to perform a meta-analysis; in this setting, its potential presence cannot be excluded.

## 5. Conclusions

In summary, the current evidence suggests a potential association between EAT and impaired MPI assessed by PET, with EAT possibly providing information that is complementary to CCS. However, the available data remain limited and heterogeneous, and further studies are needed to confirm these observations. Future research should ideally focus on prospective, multicenter studies to address the current limitations. Such studies could aim to: (1) standardize EAT quantification methods using the attenuation correction CT from PET/CT scanners; (2) evaluate the incremental prognostic value of EAT metrics over established clinical risk factors, CCS, and PET perfusion parameters for predicting hard cardiac events; and (3) investigate whether interventions targeting the adipose tissue biology or volume might lead to measurable improvements in coronary vascular function, as assessed by serial PET imaging.

## Figures and Tables

**Table 1:** Characteristics of the studies considered for the review.

First Author	N. Ref.	Year	Country	Study Design	N. Pts.	Setting	Endpoint	Follow-Up
Janik M	[22]	2010	USA	Retrospective	45	CAD-naïve patients at intermediate pre-test likelihood of disease	Ischemia	na
Nakazato R	[30]	2012	USA	Retrospective	92	CAD-naïve patients	Ischemia and stenosis	6 months
Alam M	[23]	2013	Canada	Retrospective	137	CAD-naïve patients	MFR	na
Karohl C	[24]	2013	Brazil, Italy, Venezuela, USA	Retrospective	411	CKD 4–5 stage patients undergoing cardiovascular risk stratification prior to kidney transplantation	Ischemia	na
Bakkum MJ	[25]	2015	The Netherlands	Retrospective	208	CAD-naïve patients	MFR, MBF	na
Otaki Y	[31]	2015	USA, Germany	Retrospective	85	CAD-naïve patients	MFR	na
Nappi C	[26]	2019	Italy	Retrospective	270	Patients with suspected CAD and normal myocardial perfusion imaging	MFR, MBF	na
Zobel EH	[27]	2020	Denmark	Retrospective	150	Type 1 or type 2 diabetes and healthy subjects.	MFR	na
Miller RJH	[28]	2025	USA, Poland, Belgium	Retrospective	10,085	CAD-naïve and subjects with previous CAD	Outcomes (death and myocardial infarction)	na
Dondi F	[29]	2026	Italy	Retrospective	164	CAD-naïve and subjects with previous CAD	MFR, MBF	na

N.: number, Ref: reference, Pts: patients, USA: United States of America, na: not available, CAD: coronary artery disease, CKD: chronic kidney disease, MFR: myocardial flow reserve, and MBF: myocardial blood flow.

**Table 2:** Results and main findings of the studies considered for the review.

First Author	Device	Tracers	Reported Mean Activity (MBq)	Covariates for Analyses	Main Findings
Janik M	PET/CT	[82Rb]	1480–2220	Age, sex, BMI, CCS	EAT volume was an independent predictor of ischemia on PET and outperformed CCS in CAD-naïve population at intermediate pre-test probability of disease.
Nakazato R	PET/CT	[82Rb]	925–1850	Age, gender, hypertension, diabetes, hypercholesterolemia, chest pain, CCS	High EAT volume may be related to concurrent presence of both ischemia and stenosis.
Alam M	PET/CT	[82Rb]	10/kg	Age, gender, BMI, hypertension, DM2, dyslipidemia, active smoking, CCS	Increased EAT appeared to be associated with impaired MFR.
Karohl C	PET/CT	[82Rb]	1480–2220	CCS, age, gender, history of cardiovascular disease, DM, smoking	EAT and CCS may help to predict if patients waiting for kidney transplantation are at higher risk of developing abnormalities of MPI under stress.
Bakkum MJ	PET/CT	[15O] H_2_O	370	Age, gender, BMI, hypercholesterolemia, hypertension, smoking, family history, DM, CCS	EAT volume was associated with left ventricular mass independently of BMI but it was not related to coronary microvascular function.
Otaki Y	PET/CT	[82Rb]	925–1850	Age, sex, number of cardiovascular risk factors, CCS	Increased EAT volume and composite risk score combining this value with CCS significantly improve identification of impaired global MFR.
Nappi C	PET/CT	[82Rb]	1110	Age, gender, BMI, DM, hypertension, hypercholesterolemia, smoking history, family history, heart rate reserve, CCS	In patients with suspected CAD and normal MPI, EAT volume predicted hyperemic MBF and reduced MFR.
Zobel EH	PET/CT	[82Rb]	1110	na	In contrast to healthy controls, a relationship between EAT and coronary calcification or MPI type 1 or type 2 diabetes was not reported.
Miller RJH	PET/CT	[82Rb] and [13N] NH_3_	ns	SM volume and density, bone volume and density, SAT volume and density, VAT volume and density, IMAT volume and density	Higher EAT density was associated with an increased risk of death or myocardial infarction.
Dondi F	PET/CT	[13N] NH_3_	185 or 370	Age, BMI, DM, hypercholesterolemia, hypertension, smoking history, family history, gender, CAD history	Correlation with EAT volume and impaired MFR was reported.

MBq: megabecquerel, EAT: epicardial adipose tissue, MFR: myocardial flow reserve, MPI: myocardial perfusion imaging, PET/CT: positron emission tomography/computed tomography, MBF: myocardial blood flow, CAD: coronary artery disease, CCS: coronary calcium score, kg: kilogram, ns: not specified; SM: skeletal muscle; SAT: subcutaneous adipose tissue; VAT: visceral adipose tissue; IMAT: intramuscular adipose tissue; BMI: body mass index; DM: diabetes mellitus, and na: not available.

**Table 3:** Threshold and EAT segmentation methods of the studies considered for the review.

First Author	EAT	Segmentation Method	HU Thresholds	Interobserver Reproducibility
Janik M	Volume	Manual	−250 to −30	No
Nakazato R	Volume	Automated software	−190 to −30	No
Alam M	Thickness	Manual	−195 to −30	Yes
Karohl C	Volume	Manual	−250 to −30	No
Bakkum MJ	Volume	Manual	−190 to −30	Yes
Otaki Y	Volume	Automated software	−190 to −30	No
Nappi C	Volume and thickness	Manual	−190 to −30	No
Zobel EH	Volume	Automated software	−150 to −50	No
Miller RJH	Volume	Deep learning model	−190 to −30	No
Dondi F	Volume and thickness	Manual	−190 to −30	Yes

HU: Hounsfield unit and EAT: epicardial adipose tissue.

## Data Availability

No new data were created or analyzed in this study. Data supporting the reported results can be found in the PubMed/MEDLINE, Scopus, and Embase databases.

## Supplementary Materials

The following supporting information can be downloaded at: https://www.mdpi.com/article/10.3390/medsci14020194/s1, PRISMA checklist [62].
